# Supplementary material for: Resolving the full spectrum of human genome variation using Linked-Reads
Source: Genome Res. 2019 Apr;29(4):635–45. doi: 10.1101/gr.234443.118 (PMC6442396; doi:10.1101/gr.234443.118)
Supplement: Supplemental Material [file supp_gr.234443.118_Supplemental_longranger-2.2.zip › longranger-2.2.tar.bz2!/lr2.2-public/lib/stan/cmdstan/stan/doc/api/html/bfgs__linesearch_8hpp_source.html]

Stan: src/stan/optimization/bfgs\_linesearch.hpp Source File


|  |  |
| --- | --- |
|  | Stan  2.5.0  probability, sampling & optimization |


- Main Page
- Related Pages
- Namespaces
- Classes
- Files

- File List
- File Members

All Classes Namespaces Files Functions Variables Typedefs Enumerations Enumerator Friends Macros Pages

- src
- stan
- optimization

bfgs\_linesearch.hpp

Go to the documentation of this file.

1 #ifndef STAN\_\_OPTIMIZATION\_\_BFGS\_LINESEARCH\_HPP

2 #define STAN\_\_OPTIMIZATION\_\_BFGS\_LINESEARCH\_HPP

3

4 #include <cmath>

5 #include <cstdlib>

6 #include <string>

7 #include <limits>

8

9 #include <boost/math/special\_functions/fpclassify.hpp>

10

11 namespace stan {

12  namespace optimization {

34  template<typename Scalar>

35  Scalar CubicInterp(const Scalar &df0,

36  const Scalar &x1, const Scalar &f1, const Scalar &df1,

37  const Scalar &loX, const Scalar &hiX)

38  {

39  const Scalar c3((-12\*f1 + 6\*x1\*(df0 + df1))/(x1\*x1\*x1));

40  const Scalar c2(-(4\*df0 + 2\*df1)/x1 + 6\*f1/(x1\*x1));

41  const Scalar &c1(df0);

42

43  const Scalar t\_s = std::sqrt(c2\*c2 - 2.0\*c1\*c3);

44  const Scalar s1 = - (c2 + t\_s)/c3;

45  const Scalar s2 = - (c2 - t\_s)/c3;

46

47  Scalar tmpF;

48  Scalar minF, minX;

49

50  // Check value at lower bound

51  minF = loX\*(loX\*(loX\*c3/3.0 + c2)/2.0 + c1);

52  minX = loX;

53

54  // Check value at upper bound

55  tmpF = hiX\*(hiX\*(hiX\*c3/3.0 + c2)/2.0 + c1);

56  if (tmpF < minF) {

57  minF = tmpF;

58  minX = hiX;

59  }

60

61  // Check value of first root

62  if (loX < s1 && s1 < hiX) {

63  tmpF = s1\*(s1\*(s1\*c3/3.0 + c2)/2.0 + c1);

64  if (tmpF < minF) {

65  minF = tmpF;

66  minX = s1;

67  }

68  }

69

70  // Check value of second root

71  if (loX < s2 && s2 < hiX) {

72  tmpF = s2\*(s2\*(s2\*c3/3.0 + c2)/2.0 + c1);

73  if (tmpF < minF) {

74  minF = tmpF;

75  minX = s2;

76  }

77  }

78

79  return minX;

80  }

81

103  template<typename Scalar>

104  Scalar CubicInterp(const Scalar &x0, const Scalar &f0, const Scalar &df0,

105  const Scalar &x1, const Scalar &f1, const Scalar &df1,

106  const Scalar &loX, const Scalar &hiX)

107  {

108  return x0 + CubicInterp(df0,x1-x0,f1-f0,df1,loX-x0,hiX-x0);

109  }

110

111  namespace {

115  template<typename FunctorType, typename Scalar, typename XType>

116  int WolfLSZoom(Scalar &alpha, XType &newX, Scalar &newF, XType &newDF,

117  FunctorType &func,

118  const XType &x, const Scalar &f, const Scalar &dfp,

119  const Scalar &c1dfp, const Scalar &c2dfp, const XType &p,

120  Scalar alo, Scalar aloF, Scalar aloDFp,

121  Scalar ahi, Scalar ahiF, Scalar ahiDFp,

122  const Scalar &min\_range)

123  {

124  Scalar d1, d2, newDFp;

125  int itNum(0);

126

127  while (1) {

128  itNum++;

129

130  if (std::fabs(alo-ahi) < min\_range)

131  return 1;

132

133  if (itNum%5 == 0) {

134  alpha = 0.5\*(alo+ahi);

135  }

136  else {

137  // Perform cubic interpolation to determine next point to try

138  d1 = aloDFp + ahiDFp - 3\*(aloF-ahiF)/(alo-ahi);

139  d2 = std::sqrt(d1\*d1 - aloDFp\*ahiDFp);

140  if (ahi < alo)

141  d2 = -d2;

142  alpha = ahi - (ahi - alo)\*(ahiDFp + d2 - d1)/(ahiDFp - aloDFp + 2\*d2);

143  if (!boost::math::isfinite(alpha) ||

144  alpha < std::min(alo,ahi)+0.01\*std::fabs(alo-ahi) ||

145  alpha > std::max(alo,ahi)-0.01\*std::fabs(alo-ahi))

146  alpha = 0.5\*(alo+ahi);

147  }

148

149  newX = x + alpha\*p;

150  while (func(newX,newF,newDF)) {

151  alpha = 0.5\*(alpha+std::min(alo,ahi));

152  if (std::fabs(std::min(alo,ahi)-alpha) < min\_range)

153  return 1;

154  newX = x + alpha\*p;

155  }

156  newDFp = newDF.dot(p);

157  if (newF > (f + alpha\*c1dfp) || newF >= aloF) {

158  ahi = alpha;

159  ahiF = newF;

160  ahiDFp = newDFp;

161  }

162  else {

163  if (std::fabs(newDFp) <= -c2dfp)

164  break;

165  if (newDFp\*(ahi-alo) >= 0) {

166  ahi = alo;

167  ahiF = aloF;

168  ahiDFp = aloDFp;

169  }

170  alo = alpha;

171  aloF = newF;

172  aloDFp = newDFp;

173  }

174  }

175  return 0;

176  }

177  }

178

224  template<typename FunctorType, typename Scalar, typename XType>

225  int WolfeLineSearch(FunctorType &func,

226  Scalar &alpha,

227  XType &x1, Scalar &f1, XType &gradx1,

228  const XType &p,

229  const XType &x0, const Scalar &f0, const XType &gradx0,

230  const Scalar &c1, const Scalar &c2,

231  const Scalar &minAlpha)

232  {

233  const Scalar dfp(gradx0.dot(p));

234  const Scalar c1dfp(c1\*dfp);

235  const Scalar c2dfp(c2\*dfp);

236

237  Scalar alpha0(minAlpha);

238  Scalar alpha1(alpha);

239

240  Scalar prevF(f0);

241  XType prevDF(gradx0);

242  Scalar prevDFp(dfp);

243  Scalar newDFp;

244

245  int retCode = 0, nits = 0, ret;

246

247  while (1) {

248  x1.noalias() = x0 + alpha1\*p;

249  ret = func(x1,f1,gradx1);

250  if (ret!=0) {

251  alpha1 = 0.5\*(alpha0+alpha1);

252  continue;

253  }

254  newDFp = gradx1.dot(p);

255  if ((f1 > f0 + alpha\*c1dfp) || (f1 >= prevF && nits > 0)) {

256  retCode = WolfLSZoom(alpha, x1, f1, gradx1,

257  func,

258  x0, f0, dfp,

259  c1dfp, c2dfp, p,

260  alpha0, prevF, prevDFp,

261  alpha1, f1, newDFp,

262  1e-16);

263  break;

264  }

265  if (std::fabs(newDFp) <= -c2dfp) {

266  alpha = alpha1;

267  break;

268  }

269  if (newDFp >= 0) {

270  retCode = WolfLSZoom(alpha, x1, f1, gradx1,

271  func,

272  x0, f0, dfp,

273  c1dfp, c2dfp, p,

274  alpha1, f1, newDFp,

275  alpha0, prevF, prevDFp,

276  1e-16);

277  break;

278  }

279

280  alpha0 = alpha1;

281  prevF = f1;

282  std::swap(prevDF,gradx1);

283  prevDFp = newDFp;

284

285  alpha1 \*= 10.0;

286

287  nits++;

288  }

289  return retCode;

290  }

291  }

292 }

293

294 #endif

295

stan::agrad::fabs

fvar< T > fabs(const fvar< T > &x)

**Definition:** fabs.hpp:18

boost::math::isfinite

bool isfinite(const stan::agrad::var &v)

Checks if the given number has finite value.

**Definition:** boost\_fpclassify.hpp:39

stan::optimization::WolfeLineSearch

int WolfeLineSearch(FunctorType &func, Scalar &alpha, XType &x1, Scalar &f1, XType &gradx1, const XType &p, const XType &x0, const Scalar &f0, const XType &gradx0, const Scalar &c1, const Scalar &c2, const Scalar &minAlpha)

Perform a line search which finds an approximate solution to: satisfying the strong Wolfe conditions...

**Definition:** bfgs\_linesearch.hpp:225

stan::math::max

double max(const double a, const double b)

**Definition:** max.hpp:7

stan::agrad::sqrt

fvar< T > sqrt(const fvar< T > &x)

**Definition:** sqrt.hpp:15

stan::optimization::CubicInterp

Scalar CubicInterp(const Scalar &df0, const Scalar &x1, const Scalar &f1, const Scalar &df1, const Scalar &loX, const Scalar &hiX)

Find the minima in an interval [loX, hiX] of a cubic function which interpolates the points...

**Definition:** bfgs\_linesearch.hpp:35

stan::math::e

double e()

Return the base of the natural logarithm.

**Definition:** constants.hpp:86

stan::math::min

double min(const double a, const double b)

**Definition:** min.hpp:7


---

[ Stan Home Page ]

*© 2011–2014,
Stan Development Team.*
